# Risk Factors for Bovine Tuberculosis (bTB) in Cattle in Ethiopia

**DOI:** 10.1371/journal.pone.0159083

**Published:** 2016-07-12

**Authors:** Sintayehu W. Dejene, Ignas M. A. Heitkönig, Herbert H. T. Prins, Fitsum A. Lemma, Daniel A. Mekonnen, Zelalem E. Alemu, Tessema Z. Kelkay, Willem F. de Boer

**Affiliations:** 1 Resource Ecology Group, Wageningen University, Wageningen, the Netherlands; 2 College of Agriculture and Environmental Sciences, Haramaya University, Dire Dawa, Ethiopia; 3 College of Veterinary Medicine, Haramaya University, Dire Dawa, Ethiopia; University of Minnesota, UNITED STATES

## Abstract

Bovine tuberculosis (bTB) infection is generally correlated with individual cattle’s age, sex, body condition, and with husbandry practices such as herd composition, cattle movement, herd size, production system and proximity to wildlife—including bTB maintenance hosts. We tested the correlation between those factors and the prevalence of bTB, which is endemic in Ethiopia’s highland cattle, in the Afar Region and Awash National Park between November 2013 and April 2015. A total of 2550 cattle from 102 herds were tested for bTB presence using the comparative intradermal tuberculin test (CITT). Data on herd structure, herd movement, management and production system, livestock transfer, and contact with wildlife were collected using semi-structured interviews with cattle herders and herd owners. The individual overall prevalence of cattle bTB was 5.5%, with a herd prevalence of 46%. Generalized Linear Mixed Models with a random herd-effect were used to analyse risk factors of cattle reactors within each herd. The older the age of the cattle and the lower the body condition the higher the chance of a positive bTB test result, but sex, lactation status and reproductive status were not correlated with bTB status. At herd level, General Linear Models showed that pastoral production systems with transhumant herds had a higher bTB prevalence than sedentary herds. A model averaging analysis identified herd size, contact with wildlife, and the interaction of herd size and contact with wildlife as significant risk factors for bTB prevalence in cattle. A subsequent Structural Equation Model showed that the probability of contact with wildlife was influenced by herd size, through herd movement. Larger herds moved more and grazed in larger areas, hence the probability of grazing in an area with wildlife and contact with either infected cattle or infected wildlife hosts increased, enhancing the chances for bTB infection. Therefore, future bTB control strategies in cattle in pastoral areas should consider herd size and movement as important risk factors.

## Introduction

Bovine tuberculosis (bTB) caused by *Mycobacterium bovis* is a zoonotic disease, and remains a cause of concern for livestock, wildlife and human health [1,2,3]. In Africa, the disease has a wide distribution with a high prevalence in both wild and domestic animals [4]. Cattle serve as the main host for *Mycobacterium bovis* worldwide [5,6,7], while other domestic animals such as pigs, cats, dogs, horses and sheep are considered to be spill-over hosts. The transmission of bTB between animals is mainly aerogenic, and close contact between animals or sharing of feed between infected and non-infected animals are major risk factors for transmission of bTB [8,9,10]. Ingestion of *M*. *bovis* from contaminated pasture or water is also a risk factor for transmission of the disease [11].

bTB outbreaks can trigger large economic costs to society since it can affect international trade of animals and animal products, create productivity losses (e.g., reduced milk yields and meat production, reduced fertility), call for expensive animal market restriction measures, trigger large control and eradication programs, and increase human health costs [1,12]. Studies found that bTB infection in cattle was associated with a 18% decrease in milk production in Bangladesh [13] and 4% in USA [14]. A study in Ireland also reported a significant difference in milk yield between bTB positive and negative cattle [15]. In developed countries, it is controlled through a test-and-slaughter policy. Nevertheless, bTB remains a problem in some countries with a well developed veterinary control system (e.g., UK, Ireland, New Zealand, USA, [2,11]), and in most developing countries, where surveillance and control activities are often inadequate or unavailable [1,12,16].

Strikingly, risk factors for bTB transmission are not well known in developing countries, as most studies were conducted in developed countries where farming practices are more intensive and control and/or eradication programmes have been implemented since decades. In Africa, most comprehensive epidemiological studies have been done in Zambia [17,18,19,20], Tanzania [21,22,23,24] and Uganda [25]. These studies have identified various risk factors for bTB transmission at different spatial levels. At individual animal level, the prevalence of tuberculosis-like lesions increased with age and decreased with increasing body condition [10,23,26]. At herd level, herd size and movement have been identified as risk factors increasing bTB transmission. In Tanzania, a high prevalence of bTB was reported in pastoral cattle with high numbers of cattle kept under intensive husbandry practice [21], whereas in Uganda, the prevalence was higher in agro-pastoral than in pastoral production systems, probably because of the closer contact between cattle and the more humid conditions in agro-pastoral systems [27]. Introduction of infected animals into the herd could also increase bTB transmission [2]. Considering the introduced animals, Reilly and Courtenay [28] demonstrated that the risk of bTB spread can be reduced by introduction of animals from a non-endemic area, minimising the number of animals introduced, and introducing more calves and yearlings than adults.

In East Africa, pastoralists keep multiple species, mainly cattle, sheep, goats, camels and donkeys, often in large herd sizes. The grazing strategy in the area relies on the movement of livestock to follow grazing and water resources over considerable distances following seasonal changes. Most studies focusing on risk factors associated with pathogens that infect multiple host species involved single species and neglected the effect of multiple hosts [4]. It is therefore necessary to explore the relation between pastoral livestock production system with transmission of bTB in a multiple host community.

Previous studies have been carried out to investigate the roles that wildlife species play on the dynamics of bTB transmission [16,29]. Wildlife hosts are classified as either spill-over hosts or maintenance hosts [2]. Spill-over hosts can be infected by bTB, and do not transmit the pathogen to other animals efficiently [3,16,29]. Increasing evidence suggests that wildlife maintenance hosts play an important role in transmission bTB to other animals [4,30]. In East Africa, humans encroach into wildlife habitats with their livestock in search of grazing areas and water, particularly during the dry season. Wildlife species that share resources with pastoralist livestock [31,32] may influence the prevalence of bTB in cattle by having direct or indirect contact (i.e., ingestion of contaminated pastures) with cattle. More studies are required to better understand the effects of interactions between ecological and animal management risk factors in multi-host communities.

Most studies focusing on ecological risk factors associated with pathogens that infect multiple host species tend to concentrate on industrialized countries, whereas the epidemiology of bTB in the developing world, especially in Africa, remains largely unknown [2]. Moreover, livestock production systems and contacts between livestock and wildlife also differ substantially between developed and developing countries [33]. bTB has been shown to be endemic in cattle from Ethiopia [34,35,36]. Given the complexity of factors affecting bTB at the individual and herd level, a study is required that quantifies the effects of multiple independent variables in a single analysis, distinguishing among the direct and indirect effects. This study therefore aimed to test which risk factors were associated with bTB prevalence in Ethiopian cattle in a pastoral area where cattle and wildlife species share grazing lands and water sources, and quantified the direct and indirect relationships between risk factors using a structural equation model.

## Materials and Methods

### Study Area

**Fig 1:**
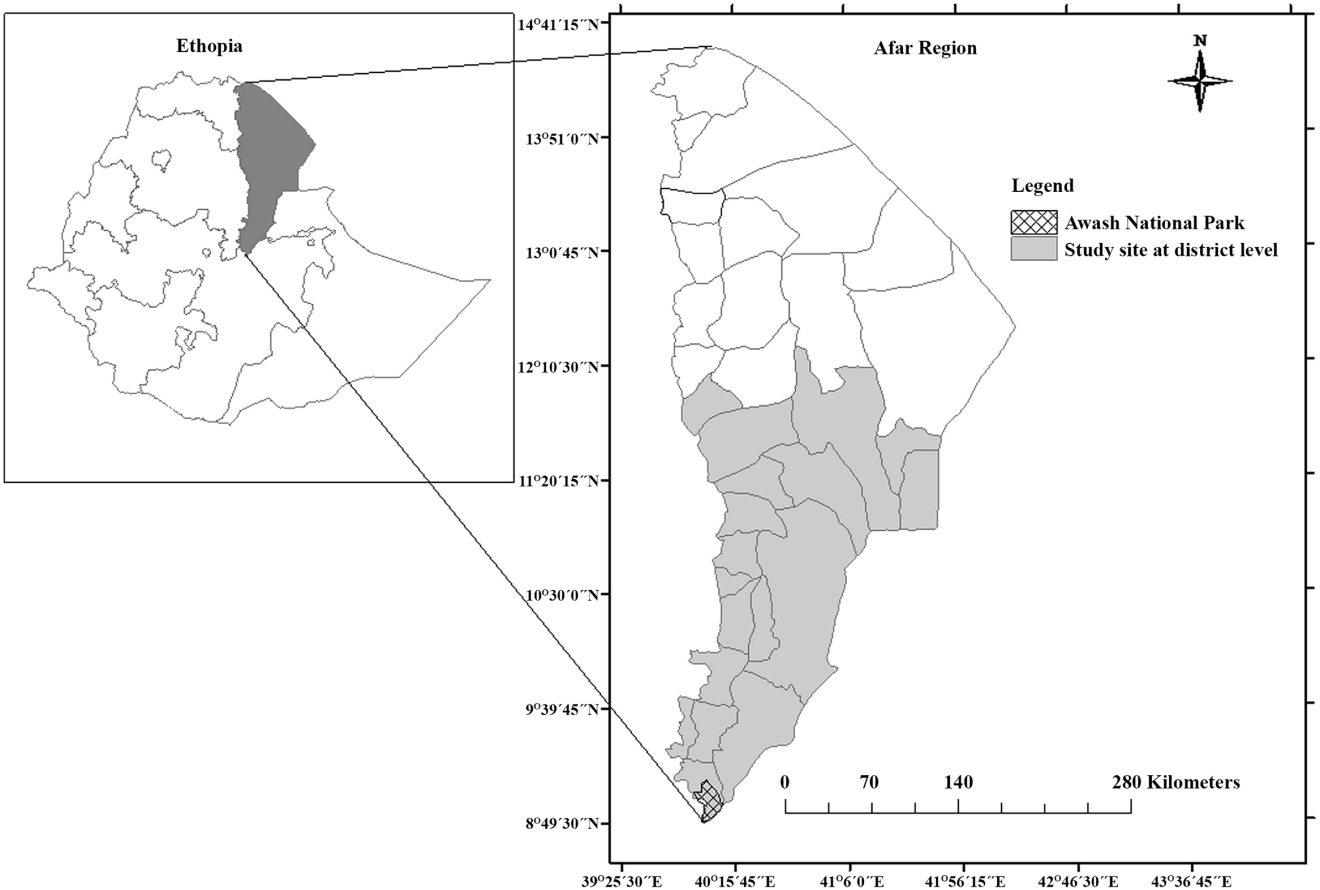
Map of the study area, the Afar Region in Ethiopia with five sub-regions (small inset) and 17 districts (larger map). The location of Awash National Park is indicated by the cross-hatched area.

The study was carried out in Awash National Park and in the neighbouring Afar Region, Ethiopia. Study sites were selected based on a gradient of wildlife-livestock interactions, livestock production systems, concentrations of livestock and wildlife, and the presence of common grazing and water resources. In the southern tip of the region, most of the grazing land and watering points are shared by livestock and wild animals from the Awash National Park (Fig 1). It is very common to observe livestock grazing in close proximity to wild animals in the study area, but wildlife-livestock co-grazing is less frequently observed when moving away from the park to the north of the study area.

Awash National Park (ANP) is located in the Ethiopian Rift valley (9°20’N, 40°20’E, 960 to 1050 m above sea level) (Fig 1) with a semi-arid savanna vegetation grazing areas. The long rainy season is from July to September and the short rainy season is from February to April. The long dry period is from October to January and the short dry period is from May to June [37]. Livestock grazing, crop production and settlement construction inside Awash National Park have become common practice in the area. Two thirds of the area demarcated as the Awash National Park is inhabited and utilized by local people for grazing of livestock in the presence of wildlife species, such greater kudu (*Tragelaphus strepsiceros*), which are known wildlife hosts of *M*. *bovis* [33,38].

The Afar region is located in the north-eastern part of Ethiopia (between 39°34’ to 42°28’E longitude and 8°49’ to 14°30’ N latitude; Fig 1) and covers about 270,000 km^2^ [39]. It is characterized by an arid and semi-arid climate with low and erratic rainfall. Rainfall is bimodal throughout the region, with a mean annual rainfall of 500 mm in the semi-arid western escarpments, decreasing to 150 mm in the arid zones to the east. The minimum and maximum annual temperature of the area is 20°C and 40°C, respectively [37]. The altitude ranges from 120 m below sea level in the Danakil depression to 1500 m above sea level. It has an estimated population of 1.5 million of which 90% are pastoralists and 10% are agro-pastoralists [40]. Afar communities traditionally keep herds of cattle, sheep, goats and camels. There are about 1.9 million cattle in the Afar region, of which 90% of the cattle are managed under a pastoral production system [40]. Afar pastoralists form a highly traditional society that has received less development attention than many comparable societies in Africa where traditional practices and institutions remain strong. In Afar society the clan is the most important political and social unit. A clan is formed by an extended group of families, and serves as a nucleus for administration and cooperation to conduct social activities among clan members. The clan is also the lowest social unit which can hold communal property rights over land and other natural resources.

### Livestock Production Systems

In the Afar region two types of pastoralism are recognised: resident or agro-pastoralists, in which animals are grazed within village perimeters without migration in search of pasture, while transhumance is the most common grazing system in the region, which involves the seasonal movements of livestock to follow suitable grazing and water resources over considerable distances in the dry season, coming back to the villages in the rainy season [41]. In the wet season, animals are kraaled at night, and in most cases herds owned by two to five close relatives or clan members are joined. The animals are released in the morning and the herds graze separately during the day in rangelands around the homesteads. In the dry season, some herds remain sedentary, whereas transhumant herds often migrate together, sharing grazing areas and watering sources along the way. Migratory distances vary from 3 to 55 km and the routes follow rivers or water sources.

### Livestock Transfer

Afar pastoralists have adapted to their harsh environment through customized social organisation. An important element of this social organisation is livestock transfer by which pastoralists may receive or bestow livestock to create and strengthen social relationships and establish trust through gifts, loans and herding contracts [42]. Livestock animals are transferred in many ways in Afar society [43,44]. The most important livestock transfer mechanism for post-crisis herd recovery is called “*iribu*”. A second important mechanism of livestock transfer is “*ala*” which is a system of bond-friendship entailing gifts of livestock. Beyond *iribu* and *ala*, animals are transferred on many other occasions, such as during a marriage, on other celebrations and ceremonies, or as compensation for injured parties.

### Study Design

A cross sectional multi-stage sampling with ‘sub-region’ as the highest stage followed by ‘district’ (n = 17), and ‘sub-district’ (n = 34) as lowest sampling stages was used to select study villages. Study animals were obtained using a three-stage random sampling procedure. The ‘village’ within the sub-district was regarded as the primary unit, the ‘herd’ as secondary unit and ‘individual animal’ as tertiary unit. Herds of livestock in each sub-district were stratified into three groups based on herd size (large, medium and small) after calculating the average herd size of the sub-district. Herds (one herd from each stratum) and individual animals were selected randomly. A total of 102 livestock herds from 34 sub-districts (3 in each sub-district, one large, medium and small herd) were selected. Informed consent was sought verbally from all livestock herders and herd owners.

### Sample Size Determination

The sample size was determined by assuming that the average expected prevalence of bTB was 11% [45]. The desired sample size was calculated using the 95% confidence interval and at 5% absolute precision following the method of Thrusfield [46]. The total sample size per district was calculated, which gave us a total number of required animals of 2550 for all 17 districts, or 75 animals for each of the 34 sub-districts. A complete list of sub-districts and villages within the sub-district was obtained from each district pastoral and agro-pastoral office. Sub-district within the district and villages within sub-district were selected using random numbers. We excluded animals younger than 1 year, since they are herded around the home-base; cows at a late stage of gestation, because they are difficult to handle, and clinically sick animals which, at late stages of the disease showed false negative responses.

### Single Comparative Intradermal Tuberculin Test

Tuberculin skin testing was performed using aliquots of 0.1 ml of 2500 IU/ml bovine purified protein derivative (PPD) and 0.1 ml of 2500 IU/ml avian PPD (Prionics Lelystad B.V, Lelystad, The Netherland). Bovine and avian PPDs were injected by veterinary staff intradermally at two sites approximately 12 centimetres apart at the border of the anterior and middle thirds of one side of the neck. This was done after shaving the two sites using a razor blade. The skin thickness was measured with digital callipers prior to and 72 h after PPD injection. An animal was considered bTB positive if the reaction at the bovine site minus the reaction at the avium site was greater than ≥ 4 mm cut-off, according to the recommendations of the World Animal Health Organization [47]. In this study, livestock owned by one owner and/or his close relatives, in which the animals shared common grazing sites, watering points, kept at night at a common site and moved together during migration, was considered as a single herd in the calculation of the herd prevalence. A herd was considered bTB positive if it had at least one tuberculin reactor animal [10,47,48]. In addition to the comparative intradermal tuberculin test, information was collected for each tested animal: sex, age, lactation and reproduction status, parity number and body condition score. Animals were categorized into three age groups: juveniles between one and three years, reproductive animals between three and ten years and animals older than ten years. Body condition of the animals was scored on a 1 (thin) to 3 (fat) scales.

### Questionnaire Survey

To identify risk factors associated with bTB prevalence, semi-structured interviews with the herders and herd owners were conducted, gathering information on general herd management practices, livestock movement and transfer, introduction of animals into the herd, pastoral production system, other livestock species kept, types and levels of herd contacts, water sources (during the wet and dry season) and contact with wild animals (Table 1). Local agricultural officers, knowledgeable on local farming practices and who had received prior training on the administration and the scope of the questions, assisted us during the interviews. All herd owners and herders of tuberculin tested cattle were interviewed by means of pre-tested questionnaires. Information on contacts between cattle herds and wildlife species was obtained from questionnaires to herders. The wildlife-livestock interaction section of the questionnaires included questions on the observation of wildlife in the grazing land and/or watering areas of cattle herds. ‘Contact with wildlife’ was defined as wildlife species being visible to the herders in the grazing and/or watering areas of cattle herds.

**Table 1:** Descriptions, abbreviations, units and summaries (mean, minimum and maximum) of the predictors used in the analysis.

Description of data sets	Predicted effect	unit
Average herd movement in a day	positive	km (7.3km)
Herd size	positive	number
Number of all new animal introduced into the herd	positive	number
Number of animals transferred due to, e.g., social relationship	positive	number
Number of sheep and goats in the herd	positive	number
Number of camels in the herd	positive	number
Number of donkeys in the herd	positive	number
Contact with wild animals	positive	class
No contact with wild animals	negative	class
Pastoral production system	positive	class
Agro-pastoral production system	negative	class
Presence of other stock	positive	class
Absent of other stock	negative	class
**Interaction terms**		
Herd size and contact with wildlife	positive	

### Herd Movement and Livestock Transfer

The interviewer estimated the maximum movement distance of the livestock herd by tracing the herd movement in the area based on interview-derived information, bound by roads, streams, rivers or hills, village, district, sub-district, or region, wildlife habitat or park, or other physical indicators, which were located on a georeferenced map. Subsequently, the maximum daily distance was calculated for sedentary and for the transhumance herds. For each herd, the total number of individuals introduced into the herd or transferred was estimated. It is likely that herds that graze close together have similar bTB prevalence, due to mixing of animals. At larger distances, herd bTB prevalence might be different, either higher or lower, due to spatial variation in bTB prevalence. To assess the impact of scale on the effect of livestock transfer on herd bTB prevalence, the effect of the number of animals introduced into the herd to strengthen social relationships was analysed separately for herds within and for herds outside the average daily herd movement radius of 7.3 km (Table 1). Livestock transfer included all animals received or bestowed to create and strengthen social relationships. Some of the Afar pastoralist kept multiple species (cattle, sheep, goat, donkey and camel), so we also considered the presence or absence of stocks other than cattle in the herd as a risk factor. Geographic coordinates and altitude were registered at the central point of each village by a global positioning system (GPS, GPSMAP 64).

### Ethical Statements

This study was approved by Haramaya University, Ethiopia (Reference number HUP14/559/15).

### Statistical Analysis

Generalized Linear Mixed Models (GLMM) were used to examine the effects of predictors on the bTB infection probability for each animal using herd as random factor, with a binary response as a dependent variable (bTB positive/negative). Different approaches were used to study the strength and the relative importance of the risk factors on bTB prevalence at herd level. Prior to developing our candidate models for the herd-level analysis, one-by-one univariate analyses were performed to identify potential risk factors, using the bTB prevalence as dependent variable in a Generalized Linear Model (GLM) (family = binomial). The number of bTB positive as well as the numbers of bTB negative cattle were specified in a two vector response variable by combining two vectors into a single object as dependent variable, comprising the bTB positive and negative cattle in a herd. Predictor variables with p<0.25 were recognized as potential risk factors, and were subsequently used to construct multiple regression models. For highly correlated independent variables, only the one causing the largest change in the log-likelihood function was added to the final global model to avoid multi-collinearity, which was assessed by checking the variance inflation factors (VIFs); the final VIF-results confirmed the absence of collinearity among explanatory variables (all VIFs<5). In addition, to investigate the effect of wildlife-livestock interactions on the prevalence of bTB, we included the interaction term between herd size and contact with wildlife after including all main factors. From the global model, candidate models were selected using ΔAIC (< 3) and Akaike weights (w>0.05), with the best approximating candidate model having the highest w, as described in Burnham and Anderson [49]. Model averaging was used to construct the final model using the Akaike weights of the different candidate models [50]. Furthermore, structural equation modelling (SEM) was conducted using the lavaan package [51,52] to study the relative direct and indirect importance of each risk factors on the bTB prevalence. In epidemiological studies, risk is often measured using different methods and metrics, and the direct pooling of regression coefficients is not meaningful to examine multivariable associations and calculate effect sizes. In such a case standardised regression coefficients offer a solution [53]. We standardized the regression coefficients so that their variances were equal to one. Thus standardised coefficients can be used as an effect size estimate when the exposure levels are measured with different units of measurement. All analysis were done using R v3.2.0.

## Results

### Animal Level Risk Factors for bTB Prevalence

The individual animal prevalence of bTB was 5.5%, whereas the herd level prevalence was 46% (47 out of 102 herds).

Risk of bTB infection increased with increasing age, as animals older than ten years had a significantly higher probability of bTB infection. There was also a strong association between having a poor body condition score and bTB infection, but sex, lactation status and reproductive status were not related with bTB status (Table 2).

**Table 2:** Summary of risk factors associated with bovine tuberculosis (bTB) in traditional Afar cattle in November 2013 to April 2015 (n = 2550).

Risk factor	Levels	Number of cattle tested	bTB reactor animals (%)	OR (95% CI)	Χ^2^	p-value
Sex	Male	272	14 (5.1)	1.0	0.1	0.770
	Female	2278	127 (5.6)	1.1 (0.62–1.92)		
Age	Juveniles	423	14 (3.3)	1.0	8.2	0.017*
	Reproductive	1776	99 (5.6)	1.7 (0.98–3.05)		
	Aged	351	28 (8.0)	2.5 (1.31–4.89)		
Lactation	Lactating	1095	54 (4.9)	1.0	1.67	0.197
	Non lactating	1183	73 (6.2)	1.3 (0.88–1.82)		
Reproduction	Gravid	821	54 (6.2)	1.0	0.95	0.377
	Non gravid	1330	73 (5.2)	1.2 (0.83–1.72)		
Body condition	Thin	414	42 (10.1)	1.0	17.6	< 0.001***
	Normal	2021	95 (4.7)	0.4 (0.30–0.64)		
	Fat	115	4 (3.5)	0.3 (0.11–0.91)		

OR = Odds Ratio, CI = 95% confidence intervals;

* *P*< 0.05;

** *p* < 0.01;

*** *p* < 0.001

### Herd Level Risk Factors for bTB Prevalence

Based on the results of the GLM analyses, seven out of 10 variables were identified as potential bTB risk factors, namely, herd size, the average herd movement in a day, number of animals introduced into the herd, number of animals transferred between herds, number of camels in a herd, pastoral production system, and contact with wildlife. These were all positively associated with bTB prevalence (Table 3). However, the number of sheep and goats, the number of donkeys, and the presence of other livestock in the herd were not correlated with bTB prevalence in cattle (Table 3).

**Table 3:** Results of the one-by-one GLM analysis of risk factors and summary statistics for all predictors against herd bTB prevalence (n = 102).

bTB prevalence				
Variables	b (95% CI)	OR(95% CI)	Χ^2^	p-value
Herd size	0.08 (0.05–0.07)	1.1 (1.04–1.06)	76.8	< 0.001***
Herd movement	0.06 (0.19–0.30)	1.2 (1.17–1.30)	63.6	< 0.001***
Number of animals introduced	0.05 (0.07–0.11)	1.1 (1.05–1.10)	36.2	< 0.001***
Number of animal transferred	0.05 (0.03–0.06)	1.0 (1.03–1.06)	24.4	< 0.001***
Number of sheep and goats	0.01 (0.01–0.02)	1.0 (0.99–1.01)	2.3	0.264
Number of donkeys	0.00 (0.09–0.11)	1.0 (0.93–1.13)	0.2	0.646
Number of camels	0.03 (0.03–0.06)	1.0 (1.03–1.06)	27.2	< 0.001***
Production system	0.06 (1.04–5.42)	11.4 (1.58–8.16)	14.7	0.016*
Contact with wildlife	0.02 (0.46–1.25)	2.0 (1.29–2.81)	9.5	<0.001***
Presence of other livestock	0.00 (0.78–1.65)	1.8 (0.57–5.79)	1.3	0.309

b = standardized regression coefficient with 95% confidence intervals, OR = Odds Ratio with 95% confidence intervals;

* *P*< 0.05;

** *p* < 0.01;

*** *p* < 0.001

Spearman's correlation matrix showed that herd movement and introduction of animals into the herd were strongly correlated with herd size and number of animals transferred, respectively (r>0.7; S1 Table), and therefore only the latter variables were included in the multiple variable model to avoid collinearity.

Variables included in the multiple linear regression analyses were herd size, number of animals transferred, number of camels, production system and contact with wildlife (S2 Table). All of these variables had a significant association with bTB prevalence in the GLM analyses (Table 3).

The model summary showed that in all candidate models and in the global model, herd size, contact with wildlife and the interaction of herd size and contact with wildlife were positively correlated to bTB prevalence and always significantly, while the effect of pastoral production system, number of camels, and transfer of livestock were not significant in all candidate models (S3 Table).

The results of model averaging showed that with increasing herd size and when the herd was in contact with wildlife the bTB prevalence increased (S1 Fig). The model also showed that the interaction of herd size and contact with wildlife had a positive effect on the prevalence of herd bTB (Table 4).

**Table 4:** Summary statistics of the final model, with standardized regression coefficient (b with 95% confidence interval), Odds Ratio (OR) with 95% confidence interval, and p-value from the GLMs for the predictors correlated with herd bTB prevalence as obtained through model averaging (n = 102).

bTB prevalence			
Variables	b (95% CI)	OR (95% CI)	*p*-value
Herd size	0.94 (0.56–1.28)	1.1 (1.04–1.09)	< 0.001***
Number of animals transferred	0.00 (0.00–0.17)	1.0 (0.96–1.01)	0.171
Number of camels in the herd	-0.11 (0.02–0.08)	1.0 (0.97–1.02)	0.246
Contact with wildlife	0.19 (0.05–0.33)	11.8 (1.43–9.64)	0.007**
Production system	0.28 (0.42–0.98)	2.3 (0.29–17.48)	0.442
Herd size and contact with wildlife	0.15 (0.02–0.04)	1.0 (0.93–0.99)	0.008**

*p<0.05;

** *p*<0.01;

*** *p*<0.001

**Fig 2:**
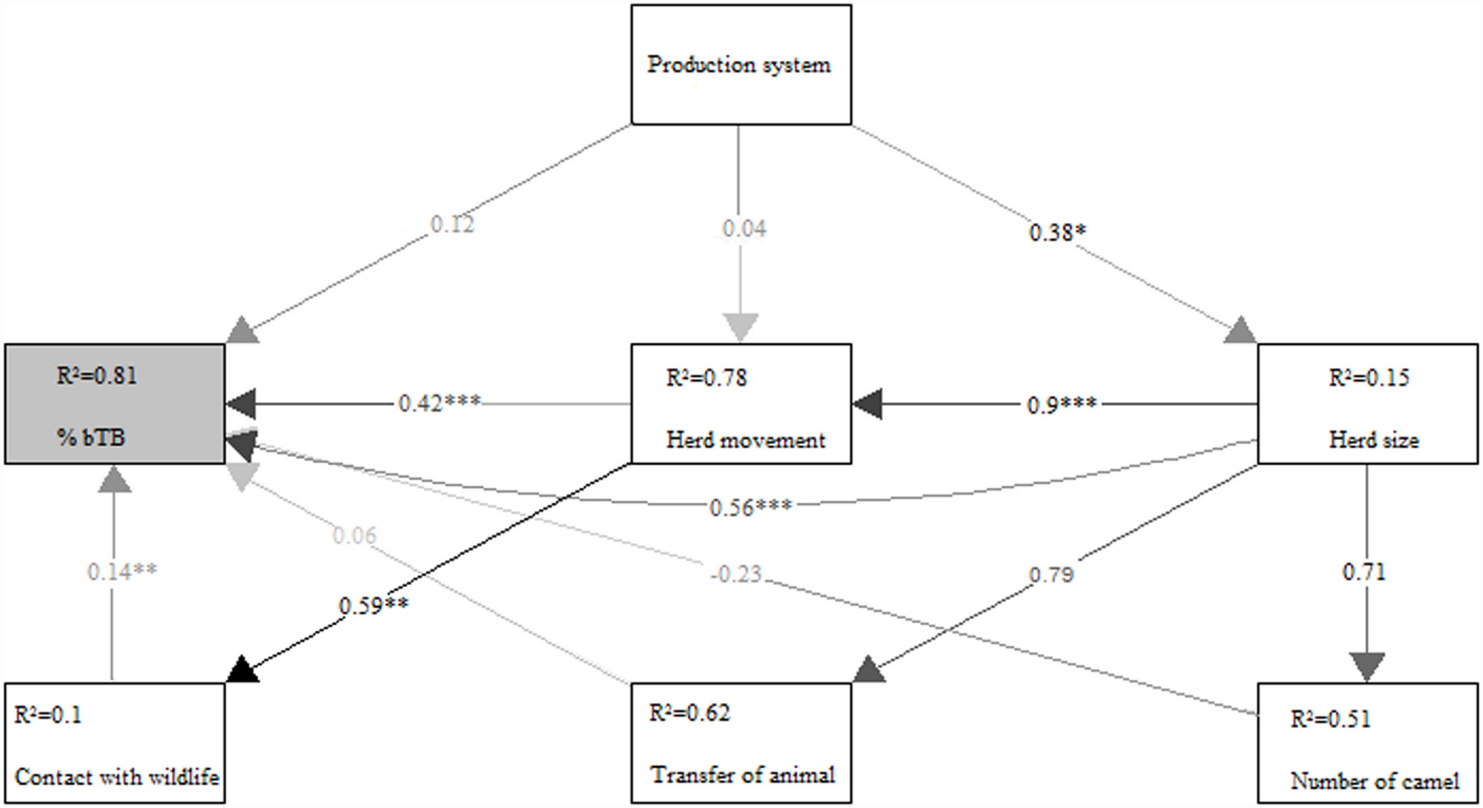
Structural equation modelling graph of the direct and indirect effects of risk factors on bTB prevalence (% bTB). The arrows indicate supported path coefficients. * *p*< 0.05; ** *p*< 0.01; *** *p*< 0.001. Variables are herd size (size), the average herd movement in a day (movement), number of animal transferred (transfer), number of camels (camel), production system (production), and contact with wildlife (wildlife). The proportion of variance explained (R^2^) appears above every response variable in the model.

The structural equation model showed that the probability of contact with wildlife, as an important risk factor for bTB infection, was mainly influenced by herd size (b_o_ = 0.9, *p*<0.001), through herd movement (b_o_ = 0.59, *p*<0.01; Fig 2).

The analysis of the impact of spatial scale on the effect of livestock transfer on herd bTB prevalence showed that introduction of animals into the herd from outside the average daily herd movement radius of 7.3 km (Table 1) reduced bTB prevalence (b = -0.11; OR = 0.89; 95% CI = 0.79–0.99; *p*<0.05) compared to an animal introduced into the herd that was obtained from a transfer within the daily herd movement radius (b = 0.04; OR = 0.96; 95% CI = 0.91–1.03; *p*>0.05).

## Discussion

The overall individual bTB prevalence was 5.5%, comparable with other results reported from Ethiopia [7,10,34,54], Uganda [55], Zambia [18,19,20] and Tanzania [56]. Our risk factor analyses identified the age of animals and body condition scores as significant factors associated with bTB infection at individual animal level. Herd size, contact with wildlife and their interaction were identified as risk factors at herd level.

In line with previous studies [7,10,45,56,57,58], bTB prevalence increased with the age of the animals, probably because of the longer exposure to the agent over time of older animals. Results also showed that a poor body condition score was associated with bTB infection. Cause and effect are not clear, however animals in poor body condition are likely more susceptible to tuberculosis infection, or tuberculosis positive animals develop a poor body condition score as a result of being infected, i.e., a clinical sign that typically follows an active infection with *M*. *bovis* [21].

Similar to what Cleaveland et al. [23] and Ameni et al. [34] found, herd size was positively correlated with the probability of bTB infection in the herd [23,34]. Transmission of bTB, which is mainly through aerosols transmission [16,59] described as a density-dependent [3]. Increasing herd size can lead to higher encounter rates of susceptible and infectious hosts, thereby promoting the spread of the pathogen within the herd. Our results support the hypothesis that herd size is a risk factor for the transmission of bTB.

Previous studies conducted in and around Awash National Park have confirmed the presence of bTB in wildlife [10]. Kafue lechwe (*K*. *leche*, [18]), greater kudu (*T*. *strepsiceros*, 5, 33), and African buffalo (*S*. *caffer*; [60]) are known as wild maintenance hosts, implicated in the transmission of *M*. *bovis* to cattle in Africa [33]. In Zambia and Tanzania high prevalence rates of bTB in cattle have been recorded within and around the wildlife area, where contact between wild maintenance hosts, particularly the lechwe and buffalo, and domestic animals were high [1,18,56]. Transmission of bTB between animals is due to direct contact when sharing forage or water resource, and/or indirect contact when grass is contaminated by infected faeces, or urine [9,61,62]. We also found that contact with wildlife was a risk factor for bTB prevalence in cattle. In the north and north eastern part of Awash National Park, it is common to observe livestock grazing in close proximity to wild animals during the dry season. In Africa, species such as greater kudu are less affected by livestock presence in their habitat use [31]. This large habitat overlap between cattle and greater kudu could play a role, and cattle could acquire bTB through grazing contaminated pastures.

**Fig 3:**
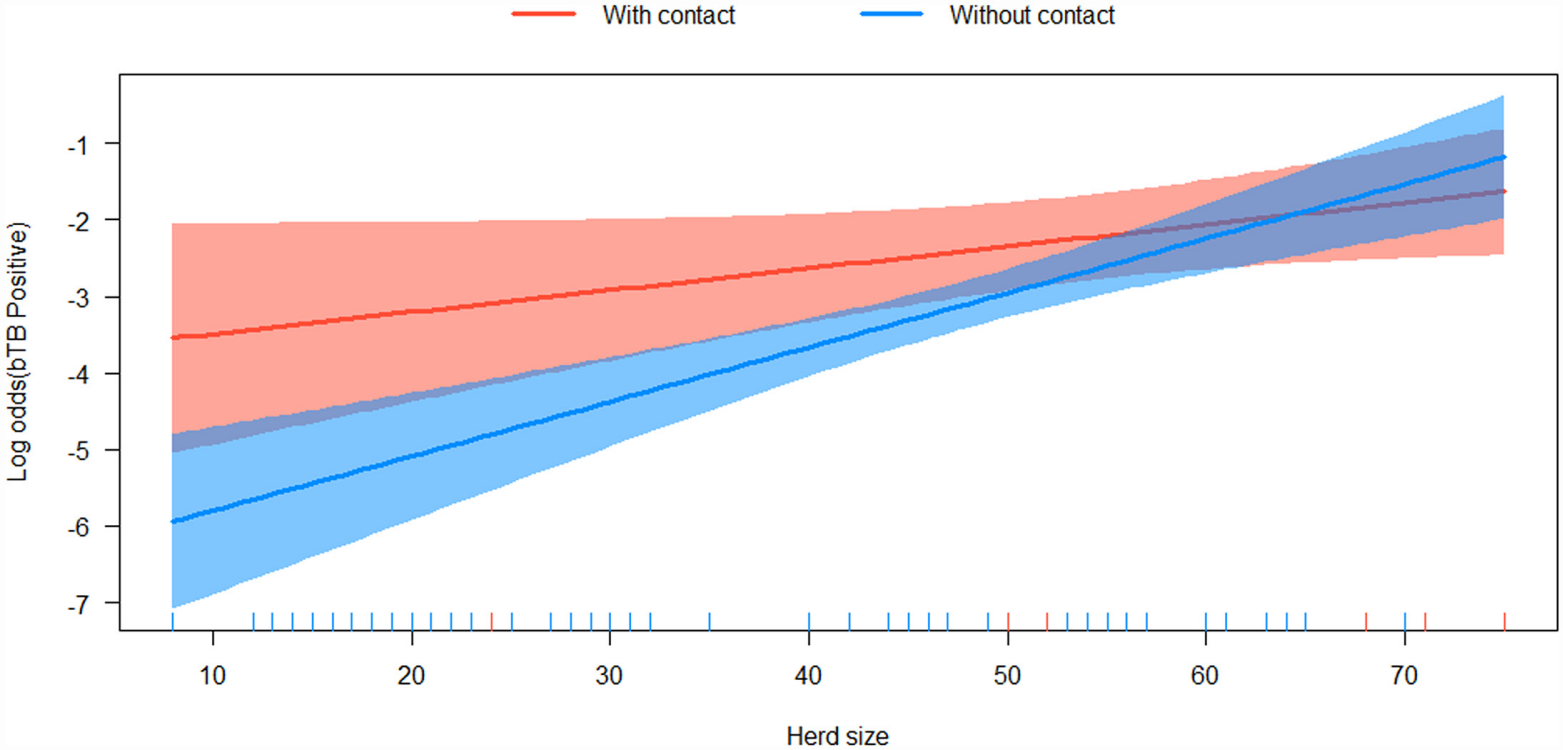
Illustration of the GLM results showing the effect of the interaction of herd size and contact with wildlife on the herd bTB prevalence (log odds scale; with 95% CLs).

The significant interaction between herd size and contact with wildlife in our study meant that the effect of contact with wildlife on the prevalence of bTB became larger when the herd size was reduced (Fig 3). However, the structural equation model showed that the probability of contact with wildlife was partly confounded with herd size, through herd movement (Fig 2). Pastoralists with larger herds move more during the dry season searching for water and pasture, and graze a larger area. These practices may predispose more cattle to bTB infection due to the higher chances of coming in contact with contaminated pastures or infected wildlife maintenance hosts. So, the correlation of contact with wildlife and bTB infection might not tell the whole story, as the underlying reasons for this contact is probably the herd size and the herd movements, which are probably more important risk factors. Moreover, we do not know to what extent wildlife is directly or indirectly infected with bTB from cattle. These uncertainties need more detailed ecological and epidemiological research.

The transfer of livestock, within and between clans, is a common practice in the area to spread risks, especially during periods of drought and/or conflicts. We found that transfer of animals was not positively correlated with bTB prevalence. Introduction of an infected animal into a bTB free herd or area is one of the major risk factors for introducing the disease [62,63,64]. We found that the number of animals introduced into the herd outside the average daily herd movement radius (7.3 km, Table 4) reduced bTB prevalence. This could be due to the fact that, within a village, cattle herds share water holes and grazing areas with each other, and come in close contact with one another; this homogenizes the infection prevalence of bTB among cattle, resulting in a similar herd infection prevalence across village herds. Similarly, Others found that introduction of animals from non-endemic area, or minimizing the number of animals being introduced, and introducing juveniles lowered the risk of bTB spread [28]. Therefore, we conclude that whether the transfer of livestock either positively or negatively affects bTB prevalence depends on the status of the transferred animals, the number transferred, the age of the animal, the distances between receivers and donors, and the spatial variation of the bTB prevalence of the herds from which animals are received.

Two types of production and grazing system are practiced in the area, i.e., village resident herds with an agro-pastoral production system, and transhumant herds, following a pastoral production system. bTB prevalence in Uganda was higher in agro-pastoral production systems of Uganda [28]. However, other studies indicated that a transhumant grazing system is a risk factor for infectious disease transmission [24], as the pastoral production system relies on movement of livestock following grazing and water resources over considerable distances under seasonal changes. Other studies showed that bTB occurs on both agro-pastoral and pastoral farming systems with no distinct differences in prevalence [18,21]. In line with these latter studies, our study showed that cattle under a pastoral production system showed a slightly higher, but not significant, bTB prevalence than under an agro-pastoral system. This might be explained by the interactions of the two cattle husbandry systems in the area. Transhumant movements are more intensive during the dry season, when transhumant herds graze in farmlands or on cotton farms. During these moments there is a lot of interaction between agro-pastoral and the pastoral cattle on these grazing lands, possibly resulting in a similar infection prevalence.

In conclusion, this study identified that bTB prevalence increased with increasing age of cattle and with decreasing body condition at individual animal level. Herd size is an important risk factor contributing to the prevalence of bTB in cattle, because larger herds have the need to move more to look for more pastures and thus end up in the wildlife conservation area where the probability of contact with wildlife maintenance hosts harbouring bTB is higher. Thus, based on the study, it is impossible to indicate the contribution of wildlife species in the transmission of bTB to cattle, and the direction of the spread of the pathogen between wildlife and cattle. Findings from this study add useful epidemiological information regarding bTB infection at the livestock-wildlife interface in Ethiopia. In order to improve this understanding, further surveillance and research on the disease ecology, including habitat use among different wildlife species with cattle, migration ecology, and population monitoring are needed.

## Supporting Information

S1 Fig Visualization of GLM results of the effects of explanatory variables on herd bTB prevalence (log odds scale with 95% CLs) in relation to herd size (A), the number of livestock transferred (B), the number of camels in the herd (C), production system (D), and contact with wildlife (E). (TIF)

S1 Questionnaire Questionnaire for (1) determining coexistence of wild and domestic herbivores in different time of the day (2) collect livestock herd information and (3) investigate direct and indirect contact between wild and domestic herbivores. (DOC)

S1 Table Spearman's correlation matrix among variables (n = 102). (DOCX)

S2 Table Summary of the global model (top) and selected candidate models (δ AIC < 3 and weight >0.05) and the variables included in the model (+ indicates the inclusion of the variable in the model). (DOCX)

S3 Table Summary statistics of the candidate models and the global model, with standardized regression coefficient (b with 95% confidence interval), Odds Ratio (OR) with 95% confidence interval, and p-value for the predictors correlated with herd bTB prevalence as obtained from GLMs (n = 102). (DOCX)
