# Correction: Risk Factors for Bovine Tuberculosis (bTB) in Cattle in Ethiopia

**DOI:** 10.1371/journal.pone.0176654

**Published:** 2017-04-20

**Authors:** Sintayehu W. Dejene, Ignas M. A. Heitkönig, Herbert H. T. Prins, Fitsum A. Lemma, Daniel A. Mekonnen, Zelalem E. Alemu, Tessema Z. Kelkay, Willem F. de Boer

There are several errors in Tables 3 and 4. Please see the corrected Tables 3 and 4 here.

**Table 3:** Results of the one-by-one GLM analysis of risk factors and summary statistics for all predictors against herd bTB prevalence (n = 102).

bTB prevalence				
Variables	b (95% CI)	OR(95% CI)	χ^2^	p-value
Herd size	0.08 (0.05–0.7)	1.1 (1.04–10.6)	76.8	< 0.001***
Herd movement	0.06 (0.01–0.30)	1.2 (1.17–1.30)	63.6	< 0.001***
Number of animals introduced	0.05 (0.007–0.11)	1.1 (1.05–1.10)	36.2	< 0.001***
Number of animal transferred	0.05 (0.03–0.06)	1.0 (-1.03–1.06)	24.4	< 0.001***
Number of sheep and goats	0.01 (0.01–0.02)	1.0 (0.99–1.01)	2.3	0.264
Number of donkeys	0.00 (-0.09–0.11)	1.0 (0.93–1.13)	0.2	0.646
Number of camels	0.03 (0.03–0.06)	1.0 (1.0–1.06)	27.2	< 0.001***
Production system	0.06 (-1.04–5.42)	11.4 (1.58–81.6)	14.7	0.016*
Contact with wildlife	0.02 (-0.46–1.25)	2.0 (1.29–2.81)	9.5	<0.001***
Presence of other livestock	0.00 (-0.78–1.65)	1.8 (0.57–5.79)	1.3	0.309

b = standardized regression coefficient with 95% confidence intervals, OR = Odds Ratio with 95% confidence intervals

* *P*< 0.05

** *p* < 0.01

*** *p* < 0.001

**Table 4:** Summary statistics of the final model, with standardized regression coefficient (b with 95% confidence interval), Odds Ratio (OR) with 95% confidence interval, and p-value from the GLMs for the predictors correlated with herd bTB prevalence as obtained through model averaging (n = 102).

bTB prevalence			
Variables	b (95% CI)	OR (95% CI)	*p*-value
Herd size	0.94 (0.56–1.28)	1.1 (1.04–10.9)	< 0.001***
Number of animals transferred	0.00 (0.00–0.17)	1.0 (0.96–1.01)	0.171
Number of camels in the herd	-0.11 (-0.20–0.08)	1.0 (-0.97–1.02)	0.246
Contact with wildlife	0.19 (0.05–0.33)	11.8 (1.43–96.4)	0.007**
Production system	0.28 (-0.42–0.98)	2.3 (0.29–17.48)	0.442
Herd size and contact with wildlife	0.15 (0.02–0.40)	1.0 (0.93–1.00)	0.008**

*p<0.05

** *p*<0.01

*** *p*<0.001
