# Supplementary figures and images for: Risk Factors for Bovine Tuberculosis (bTB) in Cattle in Ethiopia
Source: PLoS One. 2016 Jul 12;11(7):e0159083. doi: 10.1371/journal.pone.0159083 (PMC4942063; doi:10.1371/journal.pone.0159083)

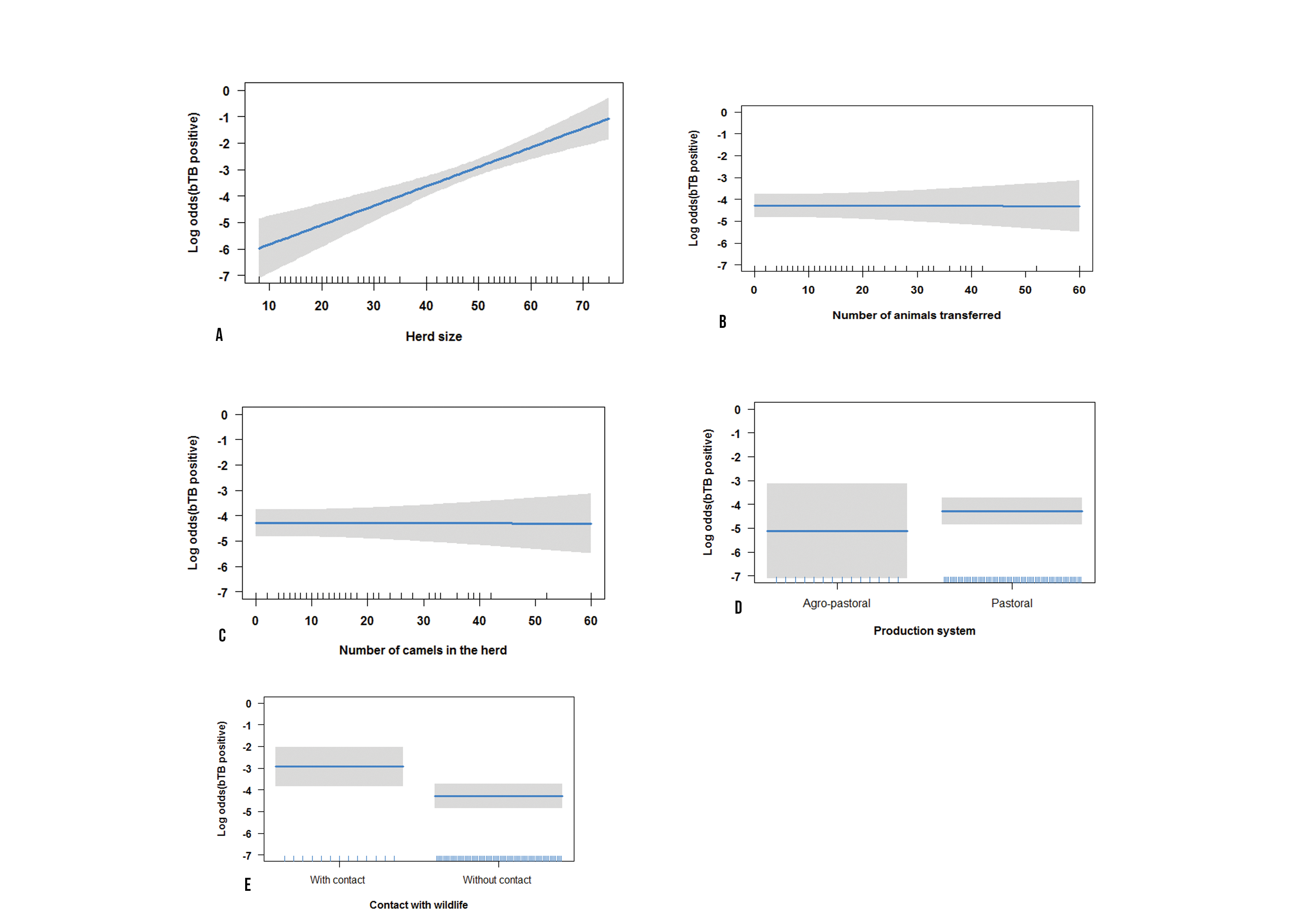

Supplement: S1 Fig — (TIF) [file pone.0159083.s001.tif]
