# Supplementary material for: Risk Factors for Bovine Tuberculosis (bTB) in Cattle in Ethiopia
Source: PLoS One. 2016 Jul 12;11(7):e0159083. doi: 10.1371/journal.pone.0159083 (PMC4942063; doi:10.1371/journal.pone.0159083)
Supplement: S1 Questionnaire — (DOC) [file pone.0159083.s002.doc]

**S1 Questionnaire**

**Questionnaire for (1) determining coexistence of wild and domestic herbivores in different time of the day (2) collect livestock herd information and (3) investigate direct and indirect contact between wild and domestic herbivores.**

**Section I Respondent characteristics**

1. Respondent code/livestock herd code______________________________
2. What are your livelihood activities? _______ 2. Livestock keeping ______ 3. Farming and livestock keeping

**Section II. Coexistence of wild and domestic herbivores**

1. For how long have you been living at this Village? _________________years
2. Have you seen the following wildlife species on your grazing or watering areas? If yes, How much/time of the day? When is the last time you saw?

| No. | Wildlife species | Yes/No | Mean group size | Last time you saw |
| --- | --- | --- | --- | --- |
| 1 | Beisa Oryx |  |  |  |
| 2 | Sommering Gazelles |  |  |  |
| 3 | Greater Kudus |  |  |  |
| 4 | Lesser Kudu |  |  |  |
| 5 | Swayne’s Hartbeest |  |  |  |
| 6 | Dik-Dik |  |  |  |
| 7 | Defarsa waterbuck |  |  |  |
| 8 | Anubis Baboon |  |  |  |
| 9 | Hamadryas Baboon |  |  |  |
| 10 | Colobus monkey |  |  |  |
| 11 | Grivet monkey |  |  |  |
| 12. | Others |  |  |  |

1. Is your livestock and wildlife use the same grazing resources? ____Yes ____No
2. Is your livestock and wildlife use the same water resources? ____Yes ____No

**Section III. Herd Information**

1. **Which species of livestock do you stock? How many number of livestock do you currently have on your herd?**

| No. | Livestock species | Number |
| --- | --- | --- |
| 1 | Cattle |  |
| 2 | Sheep |  |
| 3 | Goat |  |
| 4 | Donkey |  |
| 5 | Camel |  |
| 6 | Others |  |

1. Where do your livestock graze?

_____In side home _______ Private grazing land ___________ Communal grazing land

_____ In side wildlife habitats _________ everywhere following grazing lands

1. Where do your livestock drink water? ____________________ _______________
2. How much time it takes to travel to drinking water each day? _______________
3. How do they move, what route do they take? please describe the routes and final locations of the livestock movement____________________________________
4. Is there exchange of livestock within and between your groups clan or families? ____Yes ____No,
5. If yes, how much animals from other owners are within your herd? Where do the owners live?

| No. | Livestock species | Yes/No | How much | Since when | The Village/areas of the owners who received or give the livestock | What is the reason |
| --- | --- | --- | --- | --- | --- | --- |
| 1 | Cattle |  |  |  |  |  |
| 2 | Sheep |  |  |  |  |  |
| 3 | Goat |  |  |  |  |  |
| 4 | Donkey |  |  |  |  |  |
| 5 | Camel |  |  |  |  |  |
| 6 | Others |  |  |  |  |  |

1. How much of your own animals are grazing in other herds? Where do the herdsmen live?

| No. | Livestock species | Yes/No | How much | When | The Village/areas of the owners who received or give the livestock | What is the reason |
| --- | --- | --- | --- | --- | --- | --- |
| 1 | Cattle |  |  |  |  |  |
| 2 | Sheep |  |  |  |  |  |
| 3 | Goat |  |  |  |  |  |
| 4 | Donkey |  |  |  |  |  |
| 5 | Camel |  |  |  |  |  |
| 6 | Others |  |  |  |  |  |

1. Have you introduced livestock in to your herd from the market for the past two year?, If yes, when and how much? Where did the original owners live?

| No. | Livestock species | How much | Where is the village/area of the owner |
| --- | --- | --- | --- |
| 1 | Cattle |  |  |
| 2 | Sheep |  |  |
| 3 | Goat |  |  |
| 4 | Donkey |  |  |
| 5 | Camel |  |  |
| 6 | Others |  |  |

1. Have you sold livestock from your herd in the past two year? If yes, when and how much? Where do the buyers live?

| No. | Livestock species | How much | Where is the v  illage/area of the owner |
| --- | --- | --- | --- |
| 1 | Cattle |  |  |
| 2 | Sheep |  |  |
| 3 | Goat |  |  |
| 4 | Donkey |  |  |
| 5 | Camel |  |  |
| 6 | Others |  |  |

**Section IV. Direct and indirect contact**

- - - 1. *Do you feel that any of the following herd/animals have come into direct contact or indirect contact* *with your livestock?* Direct contact is nose-to-nose contact or sniffing, touching or licking each other. Indirect contact is eating in the same grazing land and watering from the same water points without actually touching.

| No. | Herd/animals | Physical Contact | Indirect Contact | No Contact | I Don’t Know |
| --- | --- | --- | --- | --- | --- |
|  | Livestock herds |  |  |  |  |
|  | Beisa Oryx |  |  |  |  |
|  | Sommering Gazelles |  |  |  |  |
|  | Greater Kudus |  |  |  |  |
|  | Lesser Kudu |  |  |  |  |
|  | Swayne’s Hartbeest |  |  |  |  |
|  | Dik-Dik |  |  |  |  |
|  | Defarsa waterbuck |  |  |  |  |
|  | Anubis Baboon |  |  |  |  |
|  | Hamadryas Baboon |  |  |  |  |
|  | Colobus monkey |  |  |  |  |
|  | Grivet monkey |  |  |  |  |
|  | Others |  |  |  |  |

- - - 1. If there is contact, how long do they contact/day?____________________________
