# Supplementary material for: Risk Factors for Bovine Tuberculosis (bTB) in Cattle in Ethiopia
Source: PLoS One. 2016 Jul 12;11(7):e0159083. doi: 10.1371/journal.pone.0159083 (PMC4942063; doi:10.1371/journal.pone.0159083)
Supplement: S1 Table — (DOCX) [file pone.0159083.s003.docx]

S1 Table. Spearman's correlation matrix among variables (n=102)

| Variable | Size | Movement | Introduction | Transfer | Camels | Production | Wildlife |
| --- | --- | --- | --- | --- | --- | --- | --- |
| Size | 1 |  |  |  |  |  |  |
| Movement | 0.89 | 1 |  |  |  |  |  |
| Introduction | 0.77 | 0.79 | 1 |  |  |  |  |
| Transfer | 0.69 | 0.66 | 0.84 | 1 |  |  |  |
| Camels | 0.71 | 0.69 | 0.70 | 0.62 | 1 |  |  |
| Production | 0.36 | 0.30 | 0.30 | 0.29 | 0.32 | 1 |  |
| Wildlife | 0.38 | 0.34 | 0.33 | 0.28 | 0.23 | 0.13 | 1 |

Variables are herd size (size), the average herd movement in a day (movement), number of animals introduced into the herd (introduction), number of animal transferred (transfer), number of camels (camel), production system (production), grazing system (grazing), and contact with wildlife (wildlife)
