# Supplementary material for: Risk Factors for Bovine Tuberculosis (bTB) in Cattle in Ethiopia
Source: PLoS One. 2016 Jul 12;11(7):e0159083. doi: 10.1371/journal.pone.0159083 (PMC4942063; doi:10.1371/journal.pone.0159083)
Supplement: S2 Table — (DOCX) [file pone.0159083.s004.docx]

S2 Table. Summary of the global model (top) and selected candidate models (**δ** AIC < 3 and weight >0.05) and the with variables included in the model (+ indicates the inclusion of the variable in the model).

| Model | b | Wildlife | Size | Transfer | Camel | Production | Wildlife*Size | AIC | δ AIC | weight |
| --- | --- | --- | --- | --- | --- | --- | --- | --- | --- | --- |
| Global model | -7.3 | + | + | + | + | + | + | 178.5 | 4.23 | 0.03 |
| Model 1 | -6.5 | + | + |  |  |  | + | 174.3 | 0.00 | 0.24 |
| Model 2 | -6.7 | + | + | + |  |  | + | 174.8 | 0.55 | 0.18 |
| Model 3 | -7.1 | + | + |  |  | + | + | 175.8 | 1.53 | 0.11 |
| Model 4 | -6.1 |  | + | + |  |  |  | 176.3 | 2.01 | 0.09 |
| Model 5 | -6.5 | + | + |  | + |  | + | 176.3 | 2.03 | 0.09 |
| Model 6 | -7.3 | + | + | + |  | + | + | 176.5 | 2.17 | 0.08 |
| Model 7 | -6.7 | + | + | + | + |  | + | 176.9 | 2.61 | 0.06 |
| Model 8 | -6.9 |  | + | + |  | + |  | 177.1 | 2.79 | 0.06 |
| Model 9 | -5.8 |  | + |  |  |  |  | 177.1 | 2.82 | 0.06 |

Variables are herd size (size), number of animal transferred (transfer), number of camels (camel), production system (production), contact with wildlife (wildlife), and the interaction of contact with wildlife and herd size (wildlife*size)
