# Supplementary material for: Risk Factors for Bovine Tuberculosis (bTB) in Cattle in Ethiopia
Source: PLoS One. 2016 Jul 12;11(7):e0159083. doi: 10.1371/journal.pone.0159083 (PMC4942063; doi:10.1371/journal.pone.0159083)
Supplement: S3 Table — (DOCX) [file pone.0159083.s005.docx]

S3 Table. Summary statistics of the candidate models and the global model, with standardized regression coefficient (b with 95% confidence interval), Odds Ratio (OR) with 95% confidence interval, and p-value for the predictors correlated with herd bTB prevalence as obtained from GLMs (n=102).

| Model | bTB prevalence | | | |
| --- | --- | --- | --- | --- |
|  | Variables | b (95% CI) | OR (95% CI) | p-value |
| Global model | Herd size | 0.10 (0.05-0.10) | 1.1 (1.00-1.07) | < 0.001*** |
|  | Number of animals transferred | 0.00 (0.01-0.02) | 1.0 (0.96-1.01) | 0.199 |
|  | Number of camels in the herd | -0.01(0.00-0.01) | 1.0 (0.97-1.02) | 0.624 |
|  | Contact with wildlife | 0.06 (0.45-4.60) | 8.8 (1.01-7.98) | 0.049* |
|  | Production system | 0.02 (0.79-3.72) | 2.2 (0.28-16.6) | 0.460 |
|  | Herd size and contact with wildlife | 0.06 (0.01-0.07) | 1.0 (1.00-1.07) | 0.035* |
| Model 1 | Herd size | 0.10 (0.05-0.09) | 1.1 (1.04-1.08) | < 0.001*** |
|  | Contact with wildlife | 0.07 (0.77-4.64) | 13.6 (1.74-10.6) | 0.013* |
|  | Herd size and contact with wildlife | 0.06 (0.01-0.07) | 1.0 (0.93-0.99) | 0.008** |
| Model 2 | Herd size | 0.1 (0.05-0.10) | 1.1 (1.05-1.10) | < 0.001*** |
|  | Number of animals transferred | 0.0 (0.01-0.02) | 1.0 (0.96-1.01) | 0.196 |
|  | Contact with wildlife | 0.07 (0.74-4.73) | 11.2 (1.37-9.11) | 0.024* |
|  | Herd size and contact with wildlife | 0.06 (0.01-0.07) | 1.0 (0.93-0.99) | 0.017* |
| Model 3 | Herd size | 0.10 (0.05-0.09) | 1.1 (1.04-1.08) | < 0.001*** |
|  | Contact with wildlife | 0.07 (0.71-4.63) | 11.8 (1.48-9.43) | 0.020* |
|  | Herd size and contact with wildlife | 0.06 (0.01-0.07) | 2.2 (0.28-16.5) | 0.013* |
|  | Production system | 0.02 (0.08-3.70) | 1.0 (0.93-0.99) | 0.461 |
| Model 4 | Herd size | 0.1 (0.04-0.08) | 1.1 (1.04-1.08) | < 0.001*** |
|  | Number of animals transferred | 0.0 (0.01-0.02) | 1.0 (0.96-1.00) | 0.088 |
| Model 5 | Herd size | 0.10 (0.06-0.10) | 1.1 (1.05-1.09) | < 0.001*** |
|  | Number of camels in the herd | -0.01 (0.00-0.01) | 1.0 (0.97-1.02) | 0.673 |
|  | Contact with wildlife | 0.07 (0.56-4.53) | 12.6 (1.55-10.2) | 0.018* |
|  | Herd size and contact with wildlife | 0.06 (0.01-0.07) | 1.0 (0.93-0.99) | 0.011* |
| Model 6 | Herd size | 0.10 (0.04-0.10) | 1.1 (1.05-1.09) | < 0.001*** |
|  | Number of animals transferred | 0.00 (0.00-0.02) | 1.0 (0.96-10.1) | 0.202 |
|  | Contact with wildlife | 0.07 (0.68-4.72) | 9.7 (1.17-8.11) | 0.004* |
|  | Production system | 0.02 (0.81-3.70) | 2.1 (2.74-16.2) | 0.473 |
|  | Herd size and contact with wildlife | 0.06 (0.01-0.07) | 1.0 (0.93-0.99) | 0.026* |
| Model 7 | Herd size | 0.10 (0.05-0.10) | 1.1 (1.05-1.99) | < 0.001*** |
|  | Number of animals transferred | 0.00 (0.00-0.02) | 1.0 (0.96-1.01) | 0.194 |
|  | Number of camels in the herd | -0.01 (0.00-0.01) | 1.0 (0.98-1.02) | 0.655 |
|  | Contact with wildlife | 0.07 (0.53-4.62) | 10.3 (1.23-8.73) | 0.033* |
|  | Herd size and contact with wildlife | 0.06 (0.01-0.07) | 1.1 (0.93-0.99) | 0.023* |
| Model 8 | Herd size | 0.10 (0.03-0.08) | 1.1 (1.04-1.08) | < 0.001*** |
|  | Number of animals transferred | 0.00 (0.00-0.02) | 1.0 (0.96-1.00) | 0.096 |
|  | Production system | 0.02 (0.65-3.84) | 2.8 (0.37-2.10) | 0.316 |
| Model 9 | Herd size | 0.10 (0.05-0.07) | 1.1 (1.04-1.06) | < 0.001*** |

* *P*< 0.05; ** *p* < 0.01; *** *p* < 0.001
